# RETRACTED: Abou-Donia et al. Sex-Based Differences in Plasma Autoantibodies to Central Nervous System Proteins in Gulf War Veterans versus Healthy and Symptomatic Controls. *Brain Sci.* 2021, *11*, 148

**DOI:** 10.3390/brainsci14080790

**Published:** 2024-08-05

**Authors:** Mohamed B. Abou-Donia, Maxine H. Krengel, Elizabeth S. Lapadula, Clara G. Zundel, Jessica LeClair, Joseph Massaro, Emily Quinn, Lisa A. Conboy, Efi Kokkotou, Daniel D. Nguyen, Maria Abreu, Nancy G. Klimas, Kimberly Sullivan

**Affiliations:** 1 Department of Pharmacology and Cancer Biology, Duke University Medical Center, Durham, NC 27710, USA; elizabeth.lapadula@duke.edu; 2 Department of Neurology, Boston University School of Medicine, Boston, MA 02118, USA; mhk@bu.edu (M.H.K.); cgzundel@bu.edu (C.G.Z.); 3 Department of Biostatistics, Boston University School of Public Health, Boston, MA 02118, USA; jleclai2@bu.edu (J.L.); jmm@bu.edu (J.M.); eq@bu.edu (E.Q.); 4 Department of Medicine, Harvard Medical School, Boston, MA 02115, USA; lisa_conboy@hms.harvard.edu (L.A.C.); ekokkoto@bidmc.harvard.edu (E.K.); 5 Department of Environmental Health, Boston University School of Public Health, Boston, MA 02118, USA; ddn@bu.edu; 6 Dr. Kiran C. Patel College of Osteopathic Medicine, Institute for Neuroimmune Medicine, Nova Southeastern University, Fort Lauderdale, FL 33314, USA; mariamercedesabreu@gmail.com (M.A.); nklimas@nova.edu (N.G.K.); 7 Department of Immunology, Miami VA Medical Center, Miami, FL 33125, USA

The *Brain Sciences* Editorial Office retracts the article, “Sex-Based Differences in Plasma Autoantibodies to Central Nervous System Proteins in Gulf War Veterans versus Healthy and Symptomatic Controls” [1], cited above.

Following its publication, concerns were brought to the attention of the Editorial Office by a representative from Duke University and the surviving co-authors regarding the validity of the findings of this publication [1], due to concerns related to the Western Blot images in a connected publication [2].

Adhering to our complaints procedure, an investigation was conducted by the Editorial Office and Editorial Board. Based on the review by Duke University of the article’s findings, the co-authors have requested a retraction due to concerns about their ability to rely on the Western Blot images and produce verified, reliable results. The Editorial Board evaluated the correspondence from the co-authors and the Institution and decided that the findings of this article were heavily reliant on [2], consequently deciding to retract this article [1] as per MDPI’s retraction policy (https://www.mdpi.com/ethics#_bookmark30, accessed on 28 May 2024) and in line with the Committee on Publication Ethics retraction guidelines (https://publicationethics.org/retraction-guidelines, accessed on 28 May 2024).

This retraction was approved by the Editor-in-Chief of the journal *Brain Sciences*.

The authors have agreed to this retraction.
